# Modeling Physiological Predictors of Running Velocity for Endurance Athletes

**DOI:** 10.3390/jcm11226688

**Published:** 2022-11-11

**Authors:** Szczepan Wiecha, Przemysław Seweryn Kasiak, Igor Cieśliński, Marcin Maciejczyk, Artur Mamcarz, Daniel Śliż

**Affiliations:** 1 Department of Physical Education and Health in Biala Podlaska, Faculty in Biala Podlaska, Józef Piłsudski University of Physical Education in Warsaw, 21-500 Biala Podlaska, Poland; 2 Student’s Scientific Circle of Lifestyle Medicine, 3rd Department of Internal Medicine and Cardiology, Medical University of Warsaw, 04-749 Warsaw, Poland; 3 Department of Physiology and Biochemistry, Faculty of Physical Education and Sport, University of Physical Education in Krakow, 31-571 Kraków, Poland; 4 3rd Department of Internal Medicine and Cardiology, Medical University of Warsaw, 04-749 Warsaw, Poland

**Keywords:** velocity, respiratory compensation point, anaerobic threshold, endurance training, running, prediction models, machine learning

## Abstract

**Background**: Properly performed training is a matter of importance for endurance athletes (EA). It allows for achieving better results and safer participation. Recently, the development of machine learning methods has been observed in sports diagnostics. Velocity at anaerobic threshold (V_AT_), respiratory compensation point (V_RCP_), and maximal velocity (V_max_) are the variables closely corresponding to endurance performance. The primary aims of this study were to find the strongest predictors of V_AT_, V_RCP_, V_max_, to derive and internally validate prediction models for males (1) and females (2) under TRIPOD guidelines, and to assess their machine learning accuracy. **Materials and Methods**: A total of 4001 EA (n_males_ = 3300, n_females_ = 671; age = 35.56 ± 8.12 years; BMI = 23.66 ± 2.58 kg·m^−2^; VO_2max_ = 53.20 ± 7.17 mL·min^−1^·kg^−1^) underwent treadmill cardiopulmonary exercise testing (CPET) and bioimpedance body composition analysis. XGBoost was used to select running performance predictors. Multivariable linear regression was applied to build prediction models. Ten-fold cross-validation was incorporated for accuracy evaluation during internal validation. **Results**: Oxygen uptake, blood lactate, pulmonary ventilation, and somatic parameters (BMI, age, and body fat percentage) showed the highest impact on velocity. For V_AT_ R^2^ = 0.57 (1) and 0.62 (2), derivation RMSE = 0.909 (1); 0.828 (2), validation RMSE = 0.913 (1); 0.838 (2), derivation MAE = 0.708 (1); 0.657 (2), and validation MAE = 0.710 (1); 0.665 (2). For V_RCP_ R^2^ = 0.62 (1) and 0.67 (2), derivation RMSE = 1.066 (1) and 0.964 (2), validation RMSE = 1.070 (1) and 0.978 (2), derivation MAE = 0.832 (1) and 0.752 (2), validation MAE = 0.060 (1) and 0.763 (2). For V_max_ R^2^ = 0.57 (1) and 0.65 (2), derivation RMSE = 1.202 (1) and 1.095 (2), validation RMSE = 1.205 (1) and 1.111 (2), derivation MAE = 0.943 (1) and 0.861 (2), and validation MAE = 0.944 (1) and 0.881 (2). **Conclusions**: The use of machine-learning methods allows for the precise determination of predictors of both submaximal and maximal running performance. Prediction models based on selected variables are characterized by high precision and high repeatability. The results can be used to personalize training and adjust the optimal therapeutic protocol in clinical settings, with a target population of EA.

## 1. Introduction

The benefits of regular physical exercise are widely debated and include reducing the risk of obesity [1] or cardiovascular diseases [2]. On the other hand, improperly performed training with excessive intensity may negatively affect the organism’s homeostasis and increase the risk of injury [3].

The concept of anaerobic threshold (AT) is widely discussed in exercise physiology [4]. As envisioned by Karlman Wasserman, the AT linked the increase in blood lactate concentration ([La^−^]_b_), during a strenuous incremental cardiopulmonary exercise test (CPET), with an excess arterial CO_2_ accumulation and its further pulmonary output [5]. Above the AT, [La^−^]_b_ increase leads to temporary acidosis. The endurance capacity of the whole system is usually sufficiently high to cope with the incoming state [6]. During steady-state exercise with intensity above the AT, an equilibrium in [La^−^]_b_ appearance and its elimination is observed [6]. Thus, AT is a useful practical indicator to provide personalized training recommendations (with the aim of adjusting exercise intensity to set goals) and load monitoring [7].

The respiratory compensation point (RCP) is the intensity at which arterial CO_2_ begins to decrease during demanding activity due to breathing capacity [8]. Above the RCP, the intensity of acidic ion accumulation exceeds their systemic or respiratory elimination abilities and indicates reduced endurance capacity. This leads to an over-decrease in the serum pH during graded exercise. This threshold indicates how long a high-intensity effort can be sustained [9].

The velocity at the anaerobic threshold (V_AT_), at the respiratory compensation point (V_RCP_), and at its maximum (V_max_) play an essential position in the endurance performance assessment, both for professional and recreational endurance athletes (EA), as well as for the general population under clinical conditions [10,11].

These variables are the shift points of aerobic exercise to anaerobic metabolism and can be used as one of the parameters to evaluate the maximum endurance capacity [7]. Moreover, they closely positively correlate with exercise abilities [4]. They could be incorporated into the prescription for the advancement of training plans [7] or competition strategies [12] for special and narrow populations (e.g., EA), and in sports diagnostics whenever controlled running intensity is required (i.e., in clinical CPET) [7]. Furthermore, currently, these variables most closely correspond to the EA critical power sustainability [13,14].

Apart from V_max_, maximal aerobic speed (MAS), which is directly related to VO_2max_, is another important aspect of overall performance evaluation. However, as the aim of this research is to predict V_max_, we recommend that further studies should be performed to analyze the MAS.

Numerous parameters, such as heart rate (HR), oxygen uptake (VO_2_), or anthropometric data (i.e., height, age, and gender), are widely discussed in the development of multivariable prediction models that provide an increasingly more suitable alternatives to direct CPET measurements [15].

Several studies have attempted to develop and validate various non-invasive prediction equations for different sports performance measurements (i.e., for HR, VO_2_, and others) [11,15,16]. However, they were mostly conducted on general populations or on small athletic samples, and thus, they can only be extrapolated to a low degree [17]. In addition, their methodology is widely variable, and only a few of them fulfilled recommended TRIPOD guidelines [18]. Thus, the actual number of V_AT_, V_RCP_, and V_max_ predictive models is limited, despite being significant measures of endurance capacity [19,20]. Moreover, although the variables influencing the running performance are well researched, the authors have not yet assessed how accurately they can be used to estimate running velocity by further including them in prediction models.

The aims of this study were: (1) to find the somatic and CPET variables that are the most responsible for running velocity, (2) to develop a prediction method for V_AT_, V_RCP_, and V_max_, in accordance with TRIPOD recommendations [18], (3) to internally validate the obtained formulae, (4) to assess the accuracy of the current machine-learning abilities to predict running velocity based on the primarily determined variables, and (5) to evaluate practical applications of such an approach in sports or clinical conditions based on actual knowledge regarding exercise physiology. 

## 2. Materials and Methods

The TRIPOD guidelines [18] have been applied to this research (see Supplementary Material S1, TRIPOD Checklist for Prediction Model Development and Validation). This was a retrospective data analysis from the registry of CPET performed in the years 2013–2021 at a tertiary care sports diagnostic clinic (SportsLab Clinic, Warsaw, Poland, www.sportslab.pl).

### 2.1. Ethical Approval

The study protocol was approved by the Institutional Review Board of the Bioethical Committee at the Medical University of Warsaw (AKBE/32/2021) and met the necessary regulations of the Declaration of Helsinki. Mandatory written consents to undergo incremental CPET were obtained from each EA before participating in the study. Written informed consent for participation was not required for this study, in accordance with the national legislation and the institutional requirements

### 2.2. Study Design

**Figure 1:**
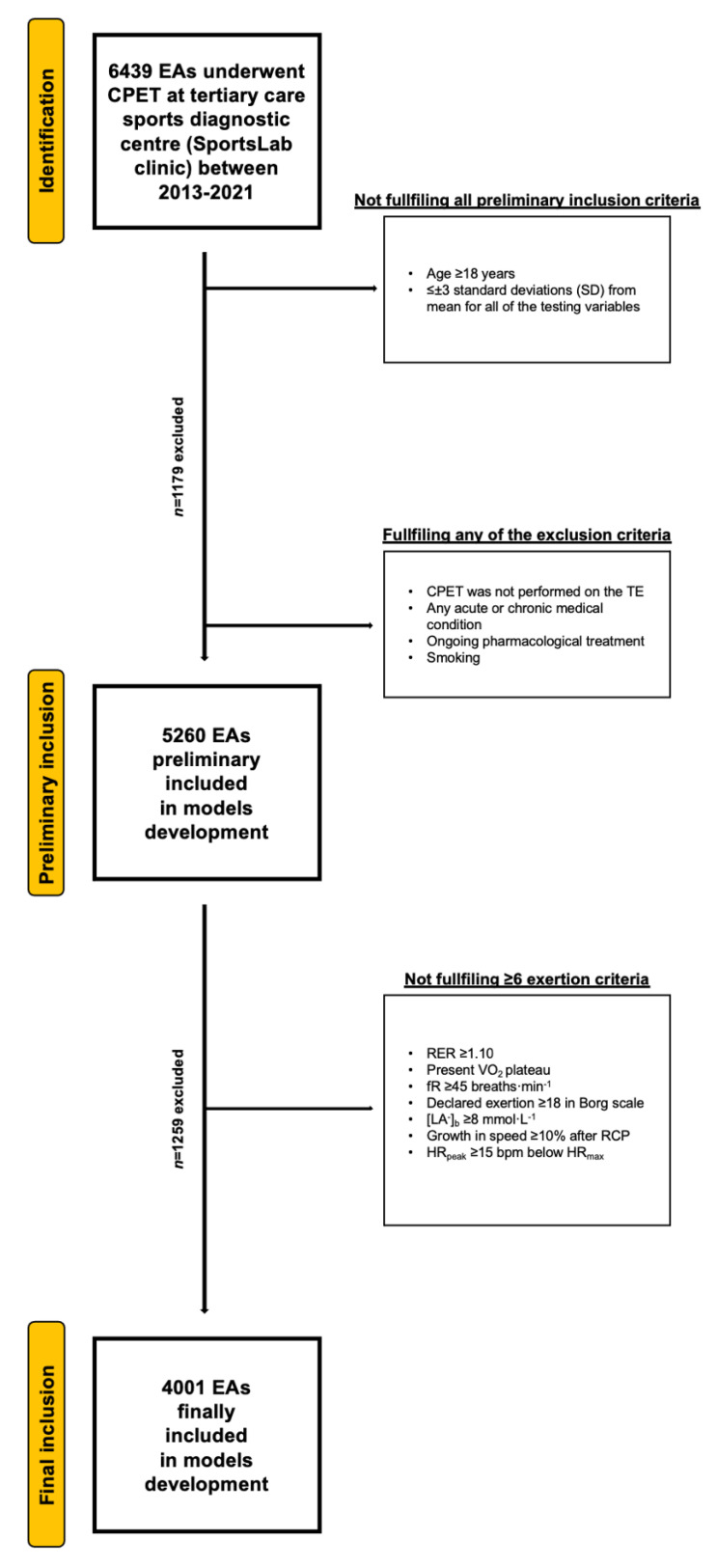
Flowchart of the preliminary inclusion and exclusion process. Abbreviations: EA, endurance athlete; CPET, cardiopulmonary exercise testing; SD, standard deviation; TE, treadmill; RER, respiratory exchange ratio; VO_2_, oxygen uptake (mL·min^−1^·kg^−1^); [La^−^]_b_, lactate concentration (mmol·L^−1^); fR, breathing frequency (breaths·min^−1^); RCP, respiratory compensation point; HR_peak_, peak heart rate (beats·min^−1^); HR_max_, maximal heart rate (bpm). At both stages of the selection, some participants met several (>1) exclusion criteria.

Participants were endurance runners who underwent CPET on the treadmill (TE). The tests were performed according to individual requests of the EA as an element of the training program prescription. Preliminary inclusion criteria were (1) age ≥ 18 years, and (2) ≤±3 SD from mean for all of the tested variables included in Table 1 (only the lowest or the weakest extreme outliers were excluded). Exclusion criteria were (1) CPET was not performed on the TE, (2) any acute or chronic medical condition (including the musculoskeletal system, or addictions), (3) ongoing pharmacological treatment, and (4) smoking. To ensure that each subject reached maximum effort during the CPET, we applied the additional selection protocol consisting of the fulfillment ≥ 6 of the following criteria: (1) plateau in VO_2_ (growth < 100 mL·min^−1^ in VO_2_ with exercise workload increase), (2) respiratory exchange ratio (RER) ≥ 1.10, (3) respiratory frequency (fR) ≥ 45 breaths·min^−1^, (4) reported exertion during CPET ≥ 18, according to the Borg scale, (5) [La^−^]_b_ ≥ 8 mmol·L^−1^, (6) increase in speed ≥ 10% of its RCP value post-exceeding the RCP, and (7) peak HR ≥ 15 beats·min^−1^ under predicted maximal HR [21]. For the entire selection procedure, along with the exclusion data, see Figure 1.

### 2.3. Somatic, [La^−^]_b_ Measurements, and CPET Protocol

First, body mass (BM) stratified by body fat (BF) and fat-free mass (FFM) measurements were obtained via the bioimpedance method (BIA) using a body composition (BC) analyzer (Tanita, MC 718, Tokyo, Japan) with the multifrequency of 5 kHz/50 kHz/250 kHz. Conditions during BC and CPET were the same: 40 m^2^ indoor, air-conditioned area, 40–60% humidity, temperature 20–22 °C, altitude 100 m ASL, and the subjects had their skin cleaned before testing. In our standardized laboratory practice, each EA had received recovery and dietary instructions via email a few days prior to testing to enable them to prepare appropriately for the CPET and BC tests. Our recommendations included: eating a high carbohydrate meal 2–3 h before the CPET and staying hydrated with sports drinks, and female EAs were advised to be well beyond their menstrual phase [22]. They also received information stating that the CPET would be performed on a mechanical TE and that they should be familiar with the characteristics of this type of effort, as well as the running technique involved.

Running tests were performed on a mechanical TE (h/p/Cosmos quasar, Nussdorf-Traunstein, Germany). CPET indices were measured using the breath-by-breath method during 15 s intervals [23], utilizing a Hans Rudolph V2 Mask (Hans Rudolph, Inc., Shawnee, KS, USA), a gas exchange analyzing device Cosmed Quark CPET (Rome, Italy), and specialized software (Quark PFT Suite powered by Omnia 10.0E). The gas analyzer device was calibrated prior to the testing protocol (16% O_2_; 5% CO_2_; ventilation accuracy ±2% or 100 mL·min^−1^). The analyzer measurement mode takes into account the manufacturer’s standard settings, i.e., 3-step smoothing and removing erroneous breaths from the analysis. HR was measured through the ANT+ and torso belt as a part of the Cosmed Quark set (accuracy similar to ECG; ±1 beats·min^−1^). [La^−^]_b_ was examined using a Super GL2 analyzer (Müller Gerätebau GmbH, Freital, Germany) employing an enzymatic-amperometric electrochemical technique. The lactate analyzer was also calibrated before each round of analysis for each participant.

CPET began with a 5 min preparatory protocol (walking or slow running at a declared “conversation” pace). The primary speed was 7–12 km·h^−1^ at a 1% inclination (the differences in the starting pace resulted from the training level of the participants and were selected on the basis of an interview on their previous sports results). The pace was increased by 1 km·h^−1^ every 2 min. VO_2_ or HR plateau (no increase in VO_2_ or HR with an increase in CPET intensity) or volitional inability to maintain intensity was the moment when the test was terminated [23,24]. Subjects were encouraged verbally to make a maximum effort. HR was considered the highest value at CPET (not averaged). Maximal VO_2_ was recorded as an average from stable VO_2_ in 10 s intervals directly before the termination of the CPET [23,24]. AT and RCP were assessed via non-direct methods based on the ventilatory concept. AT was achieved if the following measures were fulfilled: (1) VE/VO_2_ curve started to grow with the constant VE/VCO_2_ curve and (2) end-tidal partial pressure of O_2_ started to grow with the constant end-tidal partial pressure of CO_2_ [25]. RCP was achieved if the following measures were fulfilled: (1) a reduction in partial pressure of end-tidal CO_2_ (PetCO_2_) after attaining a maximal intensity; (2) a fast nonlinear growth in VE (second deflection); (3) the VE/VCO_2_ ratio achieved the lowest value and started to grow; and (4) a nonlinear growth in VCO_2_ versus VO_2_ (linearity divergence) was achieved [25]. [La^−^]_b_ was assessed by obtaining a 20 µL blood sample from a fingertip: before the test, after any speed increase, and 3 min after termination. A sample for [La^−^]_b_ analysis was taken during running without interruption or pace decrease. Each time, the sample was from the same initial puncture. The first few drops were drained onto a swab and the proper blood sample was drawn. In further analysis, the corresponding values of [La^−^]_b_ for AT, RCP, and maximal VO_2_ were determined.

### 2.4. Data Analysis and Predictors Selection

Data were saved into an Excel file (Microsoft Corporation, Redmond, WA, USA) and Python script. Further, they were calculated according to frequency (percentage) and mean (±standard deviation; SD, or 95% confidence intervals; CI) for continuous variables and the median for categorical variables. Intergroup differences (each was a continuous variable) were calculated using the Student t-test for independent variables. If there were lacking data (only for [La^−^]_b_; in 1190 cases for males and 266 cases for females in total), imputation with the random forest method (RF) was applied to fill in the gaps [26,27].

The XGBoost machine learning approach was used to select variables with the highest prediction value [28]. In order to select the variables, the population was divided into 3 groups: 60% for derivation (building group), 20% for testing (testing group), and the remaining 20% for validation (validation group). After selection, 11 variables were included in the further analysis: VO_2max_, VO_2RCP_, VO_2AT_, [La^−^]_b__RCP_, [La^−^]_b__AT_, VE_max_, VE_RCP_, age, BM, BMI, and BF. Next, selected parameters were input into multiple linear regression (MLR) modeling. As a result, only significant predictors (with *p* < 0.05) were included in the final models. The derived equations are characterized by the coefficient of determination (R^2^), root mean square error (RMSE), and mean absolute error (MAE). A 10-fold cross-validation technique [29] and the Bland–Altman plots analysis [30] were used to establish the model’s precision and accuracy during internal validation. To clarify, in the 10-fold cross-validation, the population is divided into 10 random parts. The candidate model is built on [10 − 1 = 9] training sets; then, the derived model is evaluated on the test set consisting of the remaining one part. By respectively conducting building procedures on training sets and validation on the test set 10 times, we chose the final formula with the lowest possible inaccuracy validation score (defined in this paper as the lowest RMSE and MAE) [29]. Other implemented tests to reach the complete fulfillment of MLR modeling requirements include Ramsey’s RESET test (for the correctness of specificity in MLR equations), the Chow test (for stability assessment between different coefficients), and the Durbin–Watson test (for autocorrelation of residuals). Each model was examined under the above-mentioned requirements and any irregularities have not been noted.

Our comprehensive machine learning approach enables the evaluation of each formula according to preliminary variable precision (at the stage of selection), accuracy (during model building), and recall (in internal validation).

The Ggplot 2 package (version-6.0-90; Available from: https://cran.r-project.org/web/packages/caret/index.html, accessed on 21 June 2022) in RStudio (R Core Team, Vienna, Austria; version 3.6.4), GraphPad Prism (GraphPad Software; San Diego, California USA; version 9.0.0 for Mac OS), and STATA software (StataCorp, College Station, TX, USA; version 15.1) were used for statistical analysis. A two-sided *p*-value < 0.05 was considered as the significance borderline.

## 3. Results

### 3.1. Somatic Measurements and CPET Results

The participants’ anthropometric data are presented in Table 1. The full population consisted of 4001 people, of which 3330 (83.23%) were male and 671 (16.77%) were female. All data showed a normal distribution. The mean age was 35.90 ± 8.15 years for males and 33.86 ± 7.74 years for females and the overall age ranged from 18 up to 74 years. BMI was 24.07 ± 2.44 kg·m^−2^ for men, while women had 21.64 ± 2.38 kg·m^−2^. BF percentage was relatively low, estimate at 15.49 ± 4.53 in males and 22.04 ± 5.46 in females. Significant differences between genders has been observed for height (*p* < 0.0001), BM (*p* < 0.0001), BMI (*p* < 0.0001), BF (*p* < 0.0001), and FFM (*p* < 0.0001).

CPET results are presented in Table 2. Among other measured variables, V_AT_ was 10.97 ± 1.40 km·h^−1^ and 9.64 ± 1.36 km·h^−1^ for males and females, respectively. V_RCP_ was 14.02 ± 1.74 km·h^−1^ and 12.29 ± 1.68 km·h^−1^ for males and females, respectively. The V_max_ obtained during CPET was 16.07 ± 1.93 km·h^−1^ and 14.12 ± 1.85 km·h^−1^ for males and females, respectively. The starting protocol velocity was 8.61 ± 1.28 km·h^−1^ for males and 7.60 ± 1.08 km·h^−1^ for females. When comparing both genders, significant differences (all *p* < 0.0001) were found for all the measured variables except [La^−^]_b_ at AT (*p* = 0.99), maximal respiratory exchange ratio (*p* = 0.77), and maximal HR (*p* = 0.15).

### 3.2. Prediction Models for V_AT_, V_RCP_, and V_max_

**Figure 2:**
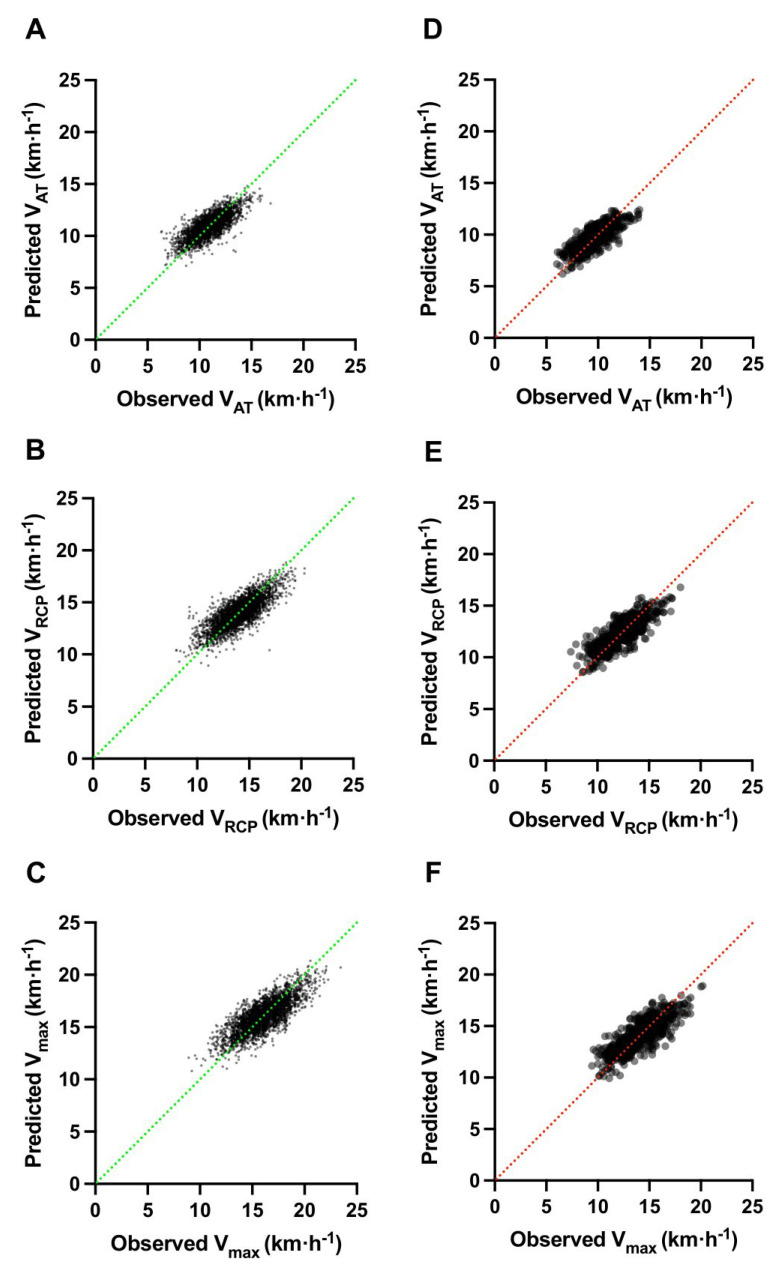
Performance of novel prediction equations for treadmill velocity. Abbreviations: V_AT_, velocity at anaerobic threshold; V_RCP_, velocity at respiratory compensation point; V_max_, maximal velocity. Colored dotted lines illustrate a 1:1 correspondence between measured and predicted velocities, respectively green for males (left row; (**A**–**C**) panels) and red for females (right row; (**D**–**F**) panels).

**Figure 3:**
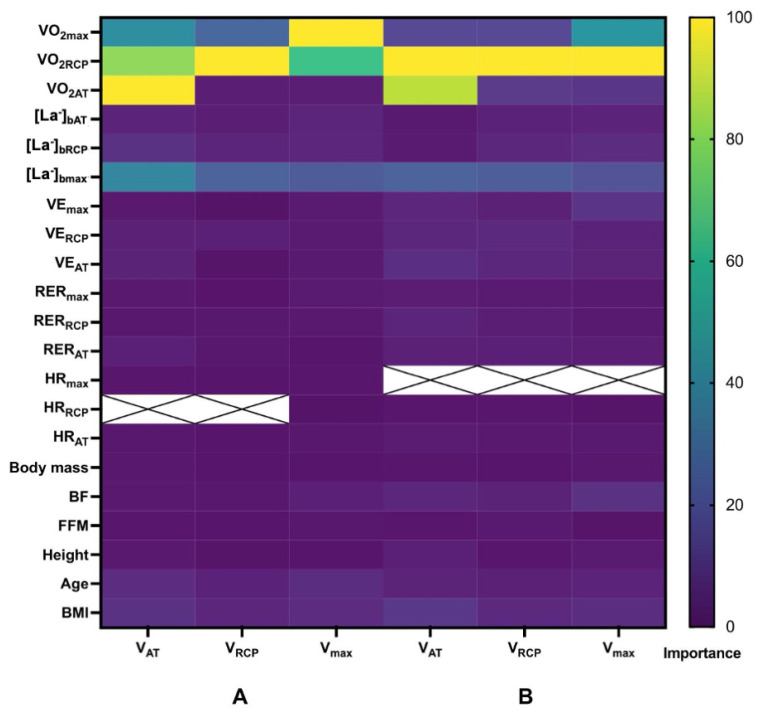
Heat map showing the importance variables regarding predicted velocity based on XGBoost selection. Abbreviation: VO_2max_, maximal oxygen uptake; VO_2RCP_, relative oxygen uptake at respiratory compensation point; VO_2AT_, relative oxygen uptake at anaerobic threshold; [La^−^]_b__RCP_, blood lactate concentration at respiratory compensation point; [La^−^]_b__AT_, blood lactate concentration at anaerobic threshold; [La^−^]_b__max_, maximal blood lactate concentration; VE_max_, maximal pulmonary ventilation; VE_RCP_, pulmonary ventilation at respiratory compensation point; VE_AT_, pulmonary ventilation at anaerobic threshold; RER_max_, maximal respiratory exchange ratio; RER_RCP_, respiratory exchange ratio at respiratory compensation point; RER_AT_, respiratory exchange ratio at anaerobic threshold; HR_max_, maximal heart rate; HR_RCP_, heart rate at respiratory compensation point; HR_AT_, heart rate at anaerobic threshold; BF, body fat; FFM, fat-free mass; BMI, body mass index; V_AT_, velocity at anaerobic threshold; V_RCP_, velocity at respiratory compensation point; V_max_, maximal velocity. Panel (**A**) presents data for males, while panel (**B**) shows the data for females. The cross means that the variable has not fulfilled preliminary selection-stage requirements (only in HR). The maps present a variable’s importance regarding the predicted velocity during the model-building stage. In the final prediction models, only the variables with significant impact (*p* < 0.05) were included.

Complete MLR prediction models for males and females are presented in Table 3 (left columns), while Figure 2 shows their performance in the derivation cohort (illustrated as an analysis of observed vs. predicted values). The importance of all CPET variables, based on XGBoost selection, included in the modeling is presented in Figure 3. The following variables showed the strongest impact in building the models: VO_2_, [La^−^], VE age, and BMI. Model performance is presented as R^2^, along with RMSE and MAE. Briefly, R^2^ for male equations ranged from 0.57 for V_AT_ and V_max_ to 0.62 for V_RCP_. For female formulae, R^2^ ranged from 0.62 for V_AT_ to 0.67 for V_RCP_. The obtained RMSE was the lowest for the female V_AT_ equation (=0.828) and the highest for the male V_max_ (=1.202), while the observed MAE was the lowest for the female V_AT_ equation (=0.657) and the highest for male V_max_ (=0.944).

### 3.3. Internal Validation

**Figure 4:**
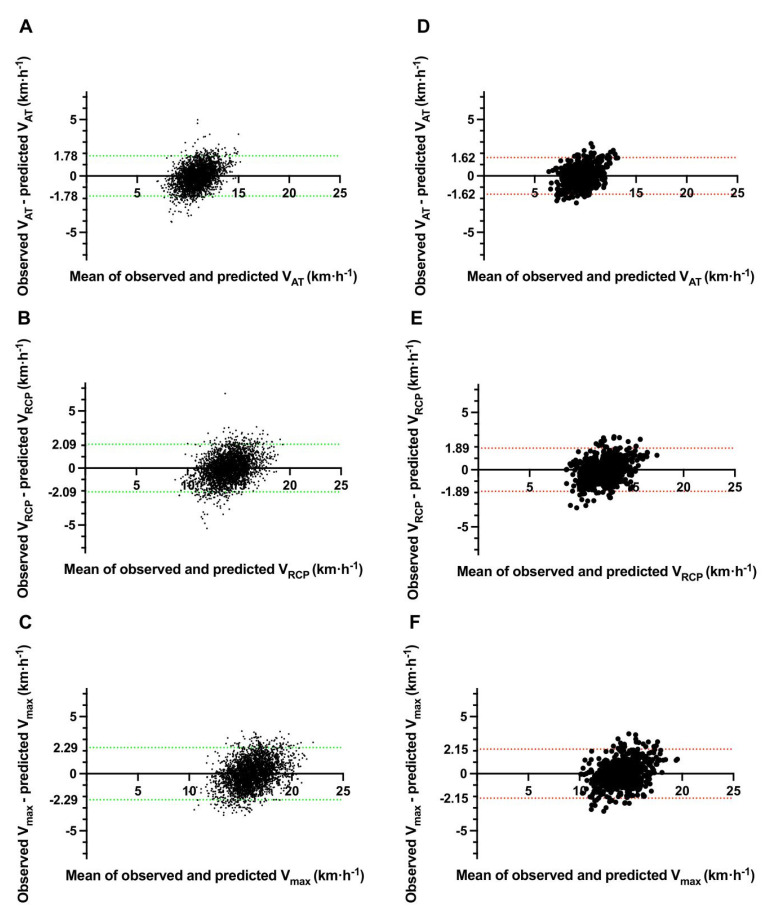
Bland–Altman Plots comparing observed with predicted velocity during internal validation. Abbreviations: V_AT_, velocity at anaerobic threshold; V_RCP_, velocity at respiratory compensation point; V_max_, maximal velocity. Colored dotted lines present a 95% confidence interval of agreement, green for males (left row; **A**–**C** panels) and red for females (right row; **D**–**F** panels), respectively.

The evaluation of each model is also presented in Table 3 (right columns). In summary, the performance of our prediction equations was similar to that observed in the derivation cohort. A slightly higher RMSE and MAE were noted. Overall, RMSE values are located between 0.838–1.205 km·h^−1^ and MAE between 0.665–0.944 km·h^−1^. The best working model (defined as having the highest replicability and the lowest risk of inaccuracies in the test set) was for V_AT_ for females (RMSE = 0.838, MAE = 0.665), and the worst was for males V_max_ (RMSE = 1.205, MAE = 0.944). The most and least accurate models were the same in regards to the derivation and validation. Figure 4 illustrates the Bland–Altman plots, with a comparison of observed vs. predicted velocity using newly derived prediction models at the stage of validation.

## 4. Discussion

In the current study, we applied advanced machine-learning properties in a comprehensive evaluation of running physiology. The obtained equations include several physiological-only measures (both anthropometric and directly measured during CPET) to provide a feasible utility for the prediction of V_AT_, V_RCP_, and V_max_ with substantial accuracy. The availability of this type of machine-learning tool in exercise diagnostics enables better training recommendations for EA and facilitates rehabilitation prescriptions for patients suffering from cardiovascular or respiratory diseases [7,31]. The novelty and main advantage are that there are no comparable studies that first select the variables with the strongest predictive abilities, and then directly evaluate their accuracy in the derived prediction models. An additional attribute is a relatively large group of healthy adult EA (*n* = 4001) who have undergone the CPET under an identical protocol, by which the maximum precision and similarity of the collected data were obtained. This enabled us to better examine whether parameters such as age [32], BC and BF [33] or VO_2max_ [16] exerted a possible significant impact on the predictive performance of the model (as they were previously classified as relevant variables in the literature. Moreover, the inclusion criteria enable us to avoid the disturbing influence of factors such as smoking [34] or medications [35].

### 4.1. Model Performance and Physiological Properties

Performance measurements show precise prediction abilities which were fairly replicable between the training and test sets (see Figure 2 and Figure 3). The obtained R^2^ explained approximately 60% to 70% of the differences, while errors were moderate-to-low, under 1 km·h^−1^ for most cases. With additional internal validation, they were both still located in the upper sensitivity range. Thus, the model accuracy was only minorly reduced. In previous publications, such as that by Petek et al. for VO_2peak_ [36], similar results were observed. However, usually, previous researchers have not carried out an initial selection of the most suitable variables, and so far, studies have been based on previously established parameters, only changing their proportions. Our study showed that VO_2_ at RCP and maximal VO_2_ were the most important parameters responsible for the prediction of middle- to long-distance running velocity (a lower impact of VO_2_ at AT was noted). This confirms previous findings by Thompson et al. and Lanferdini et al. [16,37] that the VO_2_ can be described as the universal and comparable performance measure, and that it is strongly related to running speed. Moreover, according to the physiological relationship between exercise performance and [La^−^]_b_ at AT, at RCP, and maximal VO_2_, they also significantly influence the predicted velocities (but in the varied order compared with VO_2_, with more impact from sub-maximal levels at AT or RCP than maximal [La^−^]_b_ values). This is confirmed in studies by Tanaka and Matsuura [12] and Schabort et al. [19], as growing [La^−^]_b_ and training intensity were positively correlated in both. Thus, of greater improtance seems to be the ability to rapidly utilize and prevent excess growth in [La^−^]_b_ by EA than working at maximal value for a prolonged time. Our study confirmed the previous findings by Farrell et al. [38] on this point. Another important variable was pulmonary ventilation. The majority of the influence was created by VE_RCP_, and only for V_max,_ was there a significant impact of VE_max_. The higher it was, the better running velocity was observed. Thus, it can be concluded that the higher oxygen (O_2_) supply and better carbon dioxide (CO_2_) utilization yielded an improvement in running performance. This is a well-documented concept that was stated by Sjodin and Svedenhag in the 1980s [32]. Our insights on both VO_2_ and VE also confirmed that performance at RCP is strongly correlated with other running and general exercise indices [15]. When it comes to somatic parameters, they also showed a relevant effect on velocity. Higher BMI [19] and increasing age [39] were associated with lower endurance performance. On the other hand, BC, described as a percentage of BF and FFM, showed some effect on the predicted velocity, despite their impact on males being not enough to be included in the modeling for this gender. It is worth mentioning that the influence of BF was more noticeable in females, perhaps because they naturally have a higher level of BF [40]. HR was one of the variables with the lowest impact on velocity (see Figure 3). Moreover, we emphasize that HR, which shows high inter-individual variability and is difficult to precisely estimate for EA [21], was not included in any of our equations. To summarize, the degree of the relationships between the variables is interesting. It is very promising to assess how precisely we can estimate V_AT_, V_RCP_, and V_max_ based on the above-mentioned parameters.

### 4.2. Clinical Considerations

Our results also have important clinical applications for patients from the general and athletic populations. The development of sports cardiology has resulted in a higher number of EA patients, including former cardiac patients or those suspected of having exertional cardiac abnormalities. TE CPET is often performed to some level of submaximal intensity or until refused. However, those who are less experienced may quit earlier, before reaching their optimal diagnostic intensity level, because they are not mentally adapted to perform such demanding activities [31,41]. The calculation (MET × running velocity) is used by medical professionals to provide personalized recommendations for cardiac rehabilitations [31]. Selection of the most important variables and additional comparison of those directly achieved with the predicted velocity verify whether an optimal level of intensity was achieved.

### 4.3. Practical Applications

The characteristics of selected variables and prediction models could be used in the preparation of exercise recommendations for both professional and recreational EA as patients in clinical settings [7]. The highest accuracy of the observed repeatable values would be for EA, mainly for running activities (i.e., during long-distance running or football), due to the characteristics similar to those in the derivation cohort [36]. Thanks to the use of V_AT_, V_RCP_, and V_max_ prediction models, there would be no need to run the full CPET protocol and measure all parameters, but only the most significant and contributing ones [19]. This is a matter of importance, as CPET is often impossible to perform according to the full protocol due to the limited availability of specialized clinics and equipment or the high cost of the procedure [42]. This model can also be used to verify/assess whether the athlete obtains sufficient running speed on the basis of the directly measured parameters. Of course, it currently would not be the gold standard or method of choice. Thus, results should be generalized carefully. However, they could be used as a valuable supplement to direct measurements in the present. We encourage other researchers to test our velocity prediction models and evaluate the proportion of the obtained variables using different populations to assess to what extent the results can be extrapolated and transferred.

### 4.4. Limitations

A possible limitation is that participants underwent CPET in different phases of the day (circadian rhythm), month (menstrual cycle for female athletes), or season [43,44]. Moreover, we did not evaluate the training volume of the EA. The participants received dietary and preparation tips, but we cannot be sure that they were rigorously implemented; thus, some BIA results for BC should be analyzed with caution. Some data in [La^−^]_b_ were missed (not all participants decided on the [La^−^]_b_ test because it was an optional variable in the clinic’s CPET portfolio) and RF imputation was applied. RF is recognized as the best method for filling data gaps, and our imputation did not cause a significant negative effect on the [La^−^]_b_ data precision. The models still showed high prediction abilities at the building and validation (i.e., out-of-bag error) stages. A comparison of both datasets (first set only with directly measured [La^−^]_b_ and second only with imputed [La^−^]_b_) did not show significant differences between them (*p* = 0.4) [26]. Volunteers individually declared the intensity level on the Borg scale, and the evaluation could differ between participants. The above limitations result from the specifics of the study, which is population-based, and not a controlled trial. In order to minimize their importance, the above-described internal validation was applied, which revealed the high data precision and replicability of the derived equations.

### 4.5. Future Directions

We advise that future prediction models used to estimate running velocities should be applied in cohorts with comparable characteristics to those from which they were primarily created (similar to other prediction models used in sports diagnostics) [36]. It is especially important in narrow and specified populations, including well-trained EA or cardiology patients [36]. We underline that there is a significant necessity for more accurately adjusted contributing factors and the development of new, advanced machine-learning prediction algorithms using unified TRIPOD recommendations [18]. This will enable the subsequent choice of the appropriate protocol to use in medical diagnostic and training prescriptions, depending on the participant’s disease type or fitness level [7]. We recommend assessing our methods in an external environment, such as the 3000 m distance run, to cover all evaluation sites [45,46]. It is worth mentioning that, as stated by Figueiredo et al., the critical speed showed a better predictive value for the 5 km running results regarding a steady run than the peak velocity. Although our research focuses mostly on CPET performed in the clinical settings on the mechanical treadmill, we recommend further studies which will investigate the effect of critical speed compared to peak velocity [47].

## 5. Conclusions

In summary, (1) we found the strongest predictors of running velocity, (2) we derived novel prediction models for running velocities in accordance with TRIPOD guidelines, and (3) we established their fair validation.

Currently, with the use of a machine-learning approach, we can accurately estimate V_AT_, V_RCP_, and V_max_ based only on somatic and exertion variables (the precision and repeatability in the study subgroups were comparable to the test-retest error). VO_2_, [La^−^]_b_, VE, and somatic characteristics were the greatest contributing factors. We anticipate that our findings will improve the personalization of training and rehabilitation programs. Models should be primarily applied in disciplines where running is the main form of activity, due to the similar characteristics to those regarding the specificity of the derivation cohort.

## Figures and Tables

**Table 1:** Participants’ basic anthropometric characteristics.

Variable (Unit)	Male [*n* = 3330; 83.23%]	Female [*n* = 671; 16.77%]	*p*-Value
Age (years)	35.90 (8.15)	33.86 (7.74)	<0.0001
Height (cm)	179.58 (6.22)	167.19 (6.88)	<0.0001
BM (kg)	77.72 (9.47)	60.60 (8.73)	<0.0001
BMI (kg·m^−2^)	24.07 (2.44)	21.64 (2.38)	<0.0001
BF (%)	15.49 (4.53)	22.04 (5.46)	<0.0001
FM (kg)	12.29 (4.71)	13.47 (4.65)	<0.0001
FFM (kg)	65.42 (6.47)	47.08 (6.36)	<0.0001

Abbreviations: BM, body mass; BMI, body mass index; BF, body fat; FM, fat mass; FFM, fat-free mass. The continuous value is presented as mean (SD), while the categorical value is shown as numbers (%), when appropriate. Comparisons between subgroups (*p*-value) were obtained by Student’s *t*-test for independent variables.

**Table 2:** CPET characteristics.

	Males [*n* = 3330]			Females [*n* = 671]			*p*-Value
Variable (Unit)	Mean	CI	SD	Mean	CI	SD	
VO_2AT_ (mL·min^−1^·kg^−1^)	38.42	38.25–38.59	4.96	35.69	35.32–36.05	4.83	<0.0001
VO_2AT_ (mL·min^−1^)	2955.15	2942.04–2968.26	385.79	2137.77	2113.25–2162.29	323.48	<0.0001
RER_AT_	0.87	0.87–0.87	0.04	0.86	0.85–0.86	0.04	<0.0001
HR_AT_ (beats·min^−1^)	151.32	150.96–151.68	10.70	156.45	155.66–157.24	10.39	<0.0001
VE_AT_ (L·min^−1^)	78.26	77.84–78.68	12.02	58.38	57.66–59.09	9.25	<0.0001
fR_AT_ (breaths·min^−1^)	34.88	34.61–35.14	7.85	34.89	34.31–35.47	7.66	<0.0001
[La^−^]_bAT_ (mmol·L^−1^);	1.95	1.92–1.98	0.67	1.86	1.80–1.93	0.66	0.99
VO_2RCP_ (mL·min^−1^·kg^−1^)	47.59	47.37–47.81	6.10	43.05	42.56–43.55	6.14	<0.0001
VO_2RCP_ (mL·min^−1^)	3642.72	3626.90–3658.54	465.70	2576.01	2545.15–2606.87	407.12	<0.0001
RER_RCP_	1.00	1.00–1.00	0.04	0.99	0.99–1.00	0.04	<0.0001
HR_RCP_ (beats·min^−1^)	173.43	173.12–173.75	9.33	176.04	175.34–176.73	9.12	<0.0001
VE_RCP_ (L·min^−1^)	113.82	113.25–114.39	16.43	81.15	80.20–82.11	12.34	<0.0001
fR_RCP_ (breaths·min^−1^)	44.19	43.91–44.48	8.52	43.09	42.49–43.68	7.87	<0.0001
[La^−^]_bRCP_ (mmol·L^−1^);	4.53	4.49–4.58	1.07	4.19	4.09–4.29	1.02	<0.0001
VO_2max_ (mL·min^−1^·kg^−1^)	54.10	53.87–54.34	6.93	48.73	48.23–49.24	6.67	<0.0001
VO_2max_ (mL·min^−1^)	4176.37	4157.64–4195.09	551.09	2949.02	2911.51–2986.54	494.89	<0.0001
RER_max_	1.12	1.12–1.12	0.04	1.12	1.12–1.12	0.04	0.76
HR_max_ (beats·min^−1^)	184.81	184.49–185.13	9.54	185.39	184.69–186.09	9.24	0.15
VE_max_ (L·min^−1^)	148.86	148.15–149.57	20.46	103.83	102.60–105.05	15.86	<0.0001
fR_max_ (breaths·min^−1^)	57.59	57.28–57.90	9.20	55.46	54.83–56.09	8.30	<0.0001
[La^−^]_bmax_ (mmol·L^−1^);	9.91	9.82–10.00	2.02	9.08	8.88–9.28	1.93	<0.0001
V_AT_ (km·h^−1^)	10.97	10.92–11.02	1.40	9.64	9.53–9.74	1.36	<0.0001
V_RCP_ (km·h^−1^)	14.02	13.96–14.08	1.74	12.29	12.16–12.41	1.68	<0.0001
V_max_ (km·h^−1^)	16.07	16.01–16.14	1.93	14.12	13.98–14.26	1.85	<0.0001
V_S_ (km·h^−1^)	8.61	8.56–8.66	1.28	7.60	7.51–7.69	1.08	<0.0001

Abbreviations: CI, 95% confidence interval; SD, standard deviation; VO_2AT_, oxygen uptake at anaerobic threshold; RER_AT_, respiratory exchange ratio at anaerobic threshold; HR_AT_, heart rate at anaerobic threshold; VE_AT_, pulmonary ventilation at anaerobic threshold; fR_AT_, respiratory frequency at anaerobic threshold; [La^−^]_b__AT_, lactate concentration at anaerobic threshold; VO_2RCP_, oxygen uptake at respiratory compensation point; RER_RCP_, respiratory exchange ratio at respiratory compensation point; HR_RCP_, heart rate at respiratory compensation point; VE_RCP_, pulmonary ventilation at respiratory compensation point; fR_RCP_, respiratory frequency at respiratory compensation point; [La^−^]_b__max_, lactate concentration at respiratory compensation point; VO_2max_, maximal oxygen uptake; RER_max_, maximal respiratory exchange ratio; HR_max_, maximal heart rate; VE_max_, maximal pulmonary ventilation; fR_max_, maximal respiratory frequency; [La^−^]_b__max_, maximal lactate concentration; V_ATA_, velocity at anaerobic threshold; V_RCPA_, velocity at respiratory compensation point; V_maxA_, maximal velocity; V_S_, protocol starting velocity. Comparisons between subgroups (*p*-value) were obtained by Student *t*-test for independent variables.

**Table 3:** Running velocity prediction equations stratified by gender.

Model Category	Multiple Linear Regression Equation	R^2^	Derivation Group Performance		Validation Group Performance	
			RMSE	MAE	RMSE	MAE
V_AT_ Males	8.00 − 0.01 · Age − 0.09 · BMI + 0.04 · VO_2max_ + 0.09 · VO_2AT_ − 0.65 · [La^−^]_b__AT_ + 0.01 · VE_RCP_	0.57	0.909	0.708	0.913	0.710
V_AT_ Females	7.55 − 0.02 · Age − 0.10 · BMI + 0.15 · VO_2AT_ − 0.70 · [La^−^]_b__AT_ + 0.01 · VE_RCP_	0.62	0.828	0.657	0.838	0.665
V_RCP_ Males	10.88 − 0.02 · Age − 0.11 · BMI + 0.04 · VO_2max_ − 0.99 · [La^−^]_bAT_ + 0.10 · VO_2RCP_ + 0.01 · VE_RCP_ + 0.10 · [La^−^]_bRCP_	0.62	1.066	0.832	1.070	0.835
V_RCP_ Females	9.24 − 0.02 · Age − 0.11 · BMI − 1.05 · [La^−^]_bAT_ + 0.15 · VO_2RCP_ + 0.01 · VE_RCP_ + 0.19 · [La^−^]_bRCP_	0.67	0.964	0.752	0.978	0.763
V_max_ Males	12.41 − 0.03 · Age + 0.01 · BM − 0.12 · BMI + 0.10 · VO_2max_ − 0.82 · [La^−^]_b__AT_ + 0.07 · VO_2RCP_	0.57	1.202	0.943	1.205	0.944
V_max_ Females	9.37 − 0.03 · Age + 0.06 · VO_2max_ − 0.79 · [La^−^]_bAT_ + 0.09 · VO_2RCP_ + 0.01 · VE_max_ − 0.04 · BF	0.65	1.095	0.861	1.111	0.881

Abbreviations: RMSE, root mean square error; MAE, mean absolute error; R^2^, adjusted R^2^; V_AT_, velocity at anaerobic threshold; Age, age in years; BMI, body mass index (kg·m^−2^); VO_2max_, relative maximum oxygen uptake (mL·min^−1^·kg^−1^); VO_2AT_, relative oxygen uptake at anaerobic threshold (mL·min^−1^·kg^−1^); [La^−^]_b__AT_, blood lactate concentration at anaerobic threshold (mmol·L^−1^); VE_RCP_, pulmonary ventilation at RCP (L·min^−1^); V_RCP_, velocity at respiratory compensation point; VO_2RCP_, relative oxygen uptake at respiratory compensation point (mL·min^−1^·kg^−1^); [La^−^]_b__RCP_, blood lactate concentration at respiratory compensation point; BM, body mass; V_max_, maximal velocity; VE_max_, maximal pulmonary ventilation (L·min^−1^). RMSE and MAE are explained in km·h^−1^. Model performance at the stage of derivation has been shown in the left columns. Briefly, our equations showed high accuracy and explained approximately 60–70% of the differences between participants. The results of internal validation via the 10-fold cross technique are presented in the right columns, and they showed a precise transferability, despite a limited sample size for internal validation. We are presenting one R^2^ because of the 10-fold cross-validation for the same group of participants as the derived validation.

## Data Availability

The raw data supporting the conclusions of this article will be made available by the authors, without undue reservation.

## Supplementary Materials

The following supporting information can be downloaded at: https://www.mdpi.com/article/10.3390/jcm11226688/s1, Supplementary Material S1: TRIPOD Checklist: Prediction Model Development and Validation.
